# Supplementary material for: GlycoTAIL and FlexiTAIL as Half-Life Extension Modules for Recombinant Antibody Fragments
Source: Molecules. 2022 May 19;27(10):3272. doi: 10.3390/molecules27103272 (PMC9143431; doi:10.3390/molecules27103272)
Supplement: Supplementary file 1 [file molecules-27-03272-s001.zip › molecules-1662373-supplementary.pdf]

**Supplement Figure 1:**

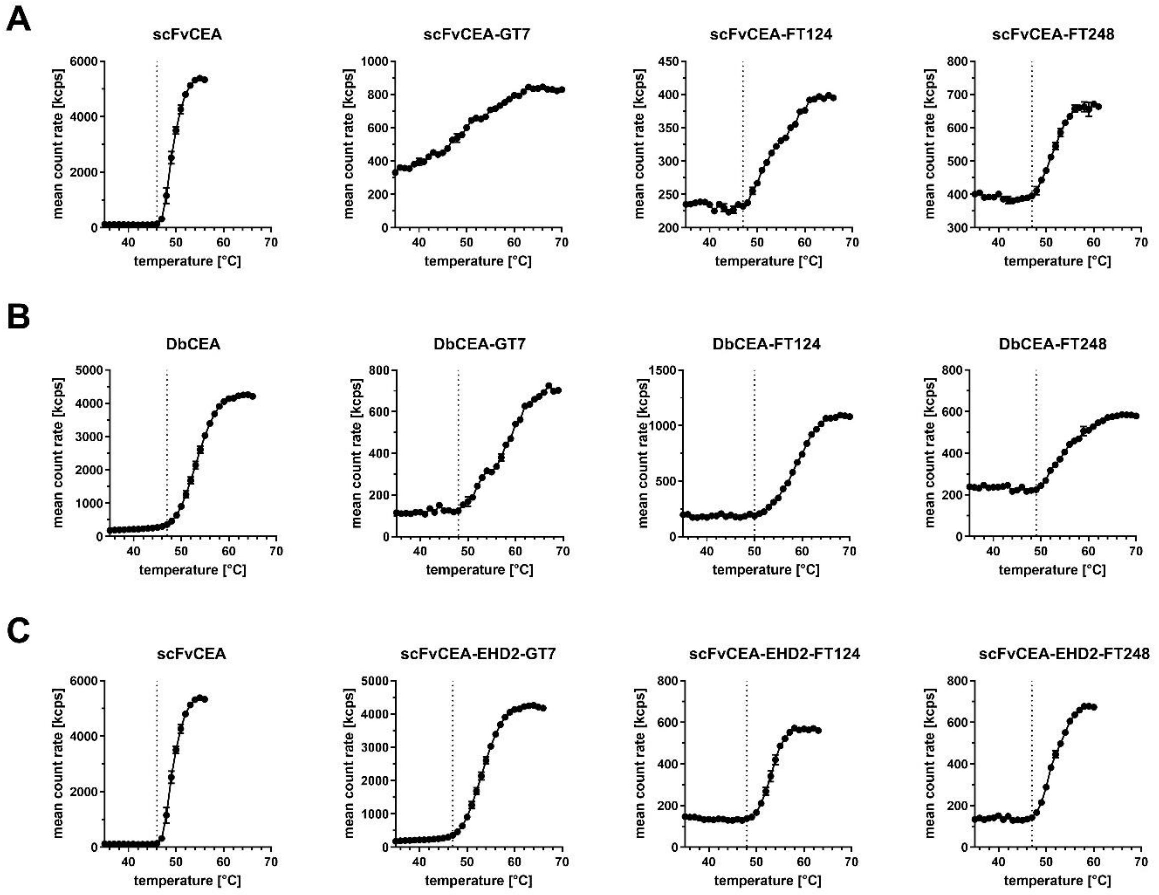


**Figure S1: Determination of aggregation point of different antibodies.** Aggregation point of the different antibodies were determined by dynamic light scattering (DLS). Aggregation points were defined as the temperature where a strong increase in signal was induced (indicated as dotted line). Determination of monovalent scFvCEA-based (**A**), of DbCEA-based (**B**), and of scFvCEA-EHD2-based (**C**) antibody molecules.
